# Gonorrhea in China: shaping medical knowledge and transforming public perceptions

**DOI:** 10.3389/fpubh.2025.1724048

**Published:** 2026-01-13

**Authors:** Peng Miao

**Affiliations:** College of Foreign Languages, University of Shanghai for Science and Technology, Shanghai, China

**Keywords:** China, gonorrhea, *Linzheng*, STIs, Traditional Chinese Medicine

## Abstract

Sexually transmitted infections (STIs) have been a persistent global challenge, with gonorrhea, caused by the bacterium *Neisseria gonorrhoeae*, remaining an enduring threat. Despite its clinical and social importance, the historical evolution of gonorrhea-related knowledge and public perceptions in China have received limited scholarly attention. This paper addresses this gap by analyzing two interconnected threads: first, the formulation of medical knowledge, encompassing Traditional Chinese Medicine’s (TCM) understanding of *linzheng* (淋证) and Western medicine’s definition of gonorrhea; second, the evolution of public perceptions, shifting from endogenous pathology to exogenous risk. This study reveals how gonorrhea transitioned from being understood as a traditional illness to being recognized as a sexually transmitted disease, from being part of general medical education to becoming a focal point of sex-oriented education, and from being perceived as an individual ailment to being understood as a social issue.

## 1 Introduction

Sexually transmitted infections (STIs) have long been recognized as a major public health challenge around the globe, carrying profound medical, social, and economic implications across human history. From ancient accounts detailing their detrimental effects on human health to contemporary efforts aimed at curbing transmission, STIs have persistently posed complex challenges to healthcare systems worldwide (1). Among the broad spectrum of STIs, gonorrhea, caused by the bacterium *Neisseria gonorrhoeae*, stands out as an enduring threat. The treatment of *N. gonorrhoeae*, like many other infectious diseases, represents one of the most successful stories of Western medicine (2). The discovery and widespread use of antibiotics have significantly reduced the burden of this disease in many parts of the world (3). Nevertheless, failure to administer appropriate antibiotic treatment or delays in intervention can cause severe and permanent health consequences (4). This highlights the importance of understanding how medical science and public opinion in different cultural contexts have responded to the introduction of Western medical approaches. In China, scholarly attention to the historical development and public understandings of STIs has predominantly centered on syphilis—specifically its translation struggles from “*yangmei chuang*” to the standardized term “*meidu*” (5). However, the conceptual evolution of gonorrhea within China’s medical discourse remains comparatively underexplored.

This article addresses this gap by analyzing two interconnected threads: first, the formulation of medical knowledge, encompassing Traditional Chinese Medicine (TCM)’s understanding of *linzheng* and Western medicine’s introduction of gonorrhea; second, the evolution of public perceptions, shifting from endogenous pathology (bodily imbalance) to exogenous risk (sexual exposure). TCM derives its knowledge primarily from classical texts, such as *Huang Di Nei Jing* (The Yellow Emperor’s Inner Classic) and *Zhubing Yuanhou Lun* (Discussions on causes and manifestations of various illnesses), which were written centuries ago, when the character “*lin* (淋)” was used to denote diseases of the urinary tract (6). Traditionally, “*relin*,” literally “heat *lin*,” was one of the five types of *linzheng* (淋证), collectively referred to as “*wulin*,” literally “five types of dripping” (7). The symptoms of *relin*, including sudden dysuria, urethral burning, chills, and pain in the lower back and lower abdomen (8), share some similarities of the symptoms of gonorrhea in Western medicine. However, it is important to note the significant differences between TCM’s understanding of urinary disorders and the modern concept of gonorrhea, despite the conceptual bridge constructed by terms like *relin* and *linzheng*.

*Linzheng*, commonly translated into “stranguria” (9), is a disease of the Kidney and Bladder, with close connections to the Heart, Liver, and Spleen (10). The explanation for *linzheng* in TCM focuses primarily on internal imbalances—such as damp-heat accumulation and the imbalance of *yin* and *yang* in the lower energizer—without addressing the nature of gonorrhea as a sexually transmitted disease (11). Conversely, Western medicine defines gonorrhea as a bacterial infection transmitted through sexual contact. It is manifest that TCM emphasizes harmonizing the internal environment, rather than eradicating specific pathogens, whereas gonorrhea in Western medicine is perceived as an STI caused by *Neisseria gonorrhoeae*, and therefore its treatment relies heavily on antibiotics to eliminate the pathogen. Such ontological discrepancy between TCM and Western medicine rendered the encounter of *relin*, a symptom-based pattern, and gonorrhea, a pathogen-defined disease, an epistemological shift in Chinese people’s understanding of urinary disorders and STIs.

## 2 Shaping medical knowledge: the encounter of *Linzheng* and gonorrhea

### 2.1 The understanding of *Linzheng* and *Relin* in TCM

The term “*lin*” appeared in early Chinese texts, such as *Shuowen Jiezi*, China’s earliest systematic etymological dictionary, where it was defined as “difficulties of urination and defecation.” In *Shenglei*, an early Chinese phonological dictionary, “*lin*” became specified as “frequent urination accompanied by difficulty in voiding.” These foundational descriptions laid the groundwork for subsequent classifications in TCM, grouping urinary disorders under the category of “*linzheng*,” or “*wulin*.” Early in *Huang Di Nei Jing*, “*lin*” was linked to urinary obstruction, as was stated in the following lines excerpted from Chapter 23 (Wide Promulgation of the Five Qi): “When the qi in the five [depots] have a disease … In the urinary bladder, if it does not pass freely it causes protuberance illness 膀胱不利为癃 (*long*); if it is unrestrained, it causes [involuntary] loss of urine (12).” Here, “protuberance illness” is probably a mistranslation due to the misunderstanding of “*long*,” which, as was explained by Fan Xingzhun (6), should be recognized as equivalent to “*lin*.” As such, the relation between the dysfunction of the bladder and *linzheng* becomes clear. Based on this, Chao Yuanfang, in *Zhubing Yuanhou Lun*, a 610 AD classic explaining the causes and symptoms of diseases, further outlined the pathogenesis of *linzheng*, attributing it to “Kidney deficiency with Bladder heat” (13). By the Tang Dynasty, *linzheng* had become classified into five subtypes, collectively known as “*wulin*.” Among these subtypes, *relin*, characterized by “urgent voiding of reddish, painful urine mixed with pus,” presents symptoms aligning with gonorrhea. From a TCM perspective, *relin* was believed to arise from severe damp-heat accumulation and heat toxicity in the urinary tract. Accordingly, treatment focused on clearing heat, resolving dampness, and restoring internal harmony. A commonly prescribed herbal formulation is Ba Zheng San, which promotes urination and alleviates pain (14).

### 2.2 The translation of gonorrhea into “*Linbing* (淋病)”

The encounter of *relin* and gonorrhea started when the latter became translated into a handful of Chinese terms, and relevant knowledge was gradually and systematically introduced to the Chinese public. The concept of gonorrhea, standardized as *linbing*, in its modern sense, was absent in TCM; in other words, the translation of gonorrhea into the Chinese language was the introduction of a novel concept. Early in Morrison’s *A Dictionary of the Chinese Language in Three Parts* published in 1822, “gonorrhea” was translated into “*baizhuo*” (15), meaning “white turbidity,” which was an established TCM term for “a mixture of urine with sperm” (7). Its meaning echoes with the Greek etymology of gonorrhea, *gonos* meaning “semen” and *rhoia* meaning “flow,” and reflects a shared understanding of pre-modern medicines. In Williams’ *An English and Chinese Vocabulary in the Court Dialect* published in 1844, “gonorrhea” was also translated into “*baizhuo*” (16). In Medhurst’s *English and Chinese Dictionary in Two Volumes* published 3 years later, “*shanqi*” was added as a second translation of “gonorrhea” (17). *Shanqi*, also a well-established TCM term meaning “elevation-illness qi” (7), is equivalent to “hernia” in modern times. The inclusion of “*shanqi*” as a translation of “gonorrhea” reflected the terminological instability and conceptual ambiguity in early practice of medical translation—problems rendered even more conspicuous when Western medical knowledge was systematically introduced to China in the mid-20th century.

Such processes of knowledge transmission were pioneered by a group of Protestant missionaries, among whom Benjamin Hobson, most famous for his translation of five medical textbooks (which were later known as “Hobsons’ Five Medical Works”), is regarded one of the most influential. In *A Medical Vocabulary in English and Chinese* published in 1858, which recorded the terminologies Hobson employed during his work, “gonorrhea,” classified as a term “used in surgery,” was translated into *liu baizhuo* (18). In this “verb + symptom” structure, “*liu*” is a common verb meaning “flowing,” which depicts continuous purulent secretion, a key symptom of gonorrhea. All of the three translations of gonorrhea were preserved in Doolittle’s *Vocabulary and Hand-Book of the Chinese Language* (19). Generally, *baizhuo* was the most common translation of gonorrhea before the 20th century; it also appeared in “Names of Diseases” in Kwong Ki-Chaou’s *An English and Chinese Dictionary* as a translation for “gonorrhea” (20).

**Figure 1:**
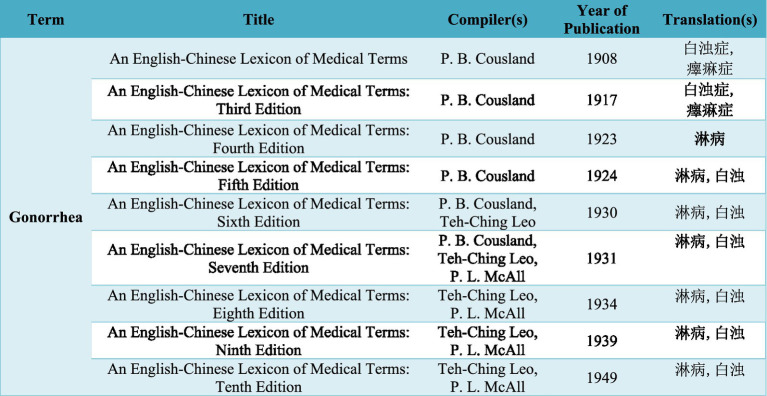
Terminological evolution of “gonorrhea” in Chinese from 1908 to 1949.

In the early 20th century, “*baizhuo*” continued to be a mainstream translation of “gonorrhea,” while more alternatives emerged. In 1904, *Technical Terms* compiled by the Committee of the Education Association of China was published, and in this lexicon, “gonorrhea” was translated into three terms—*baizhuo*, *baiyin*, and *selin* (色痳) (21), presenting an early example where the character “*lin*” (in its archaic form) was used in the translation of gonorrhea. Among the three, *baizhuo* and *baiyin* were preserved as the second and third translations of “gonorrhea” in *An English and Chinese Standard Dictionary* compiled by Yen Wei-ching, where the first option was “*linji* (痳疾)” (22). The translation “*linji*” was borrowed from the Japanese language, which was evidenced by *A Pocket Medical Lexicon*, an English-Japanese medical dictionary published in 1886, where gonorrhea was translated into “*rinshitsu* (淋疾)” (23). Different from Yen’s dictionary, Hemeling’s *English-Chinese Dictionary of the Standard Chinese Spoken Language and Handbook for Translators* preserved all of the three translations of gonorrhea in *Technical Terms*, and provided one additional option—*yilin zheng* (𤻂痳症) (24), which was borrowed from the first edition of *An English-Chinese Lexicon of Medical Terms* compiled by Cousland. In this lexicon, “gonorrhea” was translated into *baizhuo zheng* and *yilin zheng* (25). What is special about Cousland and his lexicon is that the missionary pioneered the unification of Western medical terminology in the Chinese language, which was reflected in the 10 editions of this lexicon published during 1908 and 1949. Figure 1 illustrates how the translations of gonorrhea evolved throughout the first half of the 20th century.

**Figure 2:**
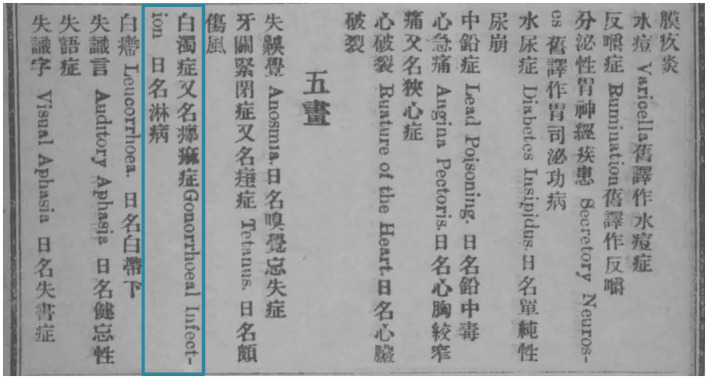
The entry of “gonorrhea” in *Zhong Wai Bing Ming Dui Zhao Biao*.

“*Linbing*,” the equivalent to gonorrhea in contemporary Chinese, emerged in the series of medical lexicons pioneered by Cousland in 1923; it was, like “*linji*,” also borrowed from the Japanese language, as evidenced by the entry of “gonorrhea” in *The New English-Japanese Dictionary* published by Sanseido in 1902 (26). In *Zhong Wai Bing Ming Dui Zhao Biao* (A Comparative Table of Chinese and Foreign Disease Names) authored by Wu Jianyuan, “*linbing*” was listed as the Japanese term for “*baizhuo zheng*,” “gonorrhoeal infection” (Figure 2) (27). This translation became recognized as a determined name for gonorrhea in *A Latin-English-German-Chinese Medical Terminology* issued by The General Committee on Scientific Terminology in 1931 (28), and served steadily as a translation of “gonorrhea” in Cousland’s lexicon since its fourth edition.

## 3 Transforming public perceptions: gonorrhea and STI awareness in China

### 3.1 The preparatory stage (before 1910)

The dissemination of gonorrhea-related knowledge in modern China can be traced back to the early 20th century, evolving through four stages—the preparatory, transition, transformation, and stabilization stages. The period before 1910 represented the first stage, during which the concept of gonorrhea initially emerged within Chinese medical discourse, and the spread of its knowledge was facilitated through two primary channels—clinical public notices and popular health lectures.

Ding Fubao, a prominent Western medical practitioner in Shanghai, played a pivotal role in popularizing Western medical knowledge during that period. His clinical notice, “A Warning to Those Suffering from Gonorrhea,” which was issued in 1909, outlined three aspects of gonorrhea-related knowledge communicated to the Chinese public: etiology, symptoms, and consequences. It was clarified that the cause of gonorrhea was “sexual intercourse with unclean women,” and thus the disease was at that time established as an STI. Ding emphasized that delayed treatment or incorrect methods would exacerbate the condition and endanger both families and society, presenting an early form of medical education regarding prevention of STIs (29).

Also published in 1909, “Popular Lecture: The Harm of Gonorrhea Transmission” introduced the concept of “*linjun* (literally bacteria of gonorrhea)”—gonococcus—discovered by Dr. Neisser in 1879, as well as explained to the Chinese public that the disease not only harmed individual health, but also caused broader social problems (30), showing that the dissemination of gonorrhea-related knowledge began to incorporate the latest findings from the West, and the perception of the disease began to shift from an individual ailment to a societal issue with public health implications.

### 3.2 The transition stage (1910–1919)

The period from 1910 to 1919 marked the transition stage in the transmission of gonorrhea-related knowledge. During this period, the understanding of gonorrhea evolved, building on the fundamental knowledge of its causative agent and transmission routes. Notably, the Chinese term “*baizhuo*” became integral to gonorrhea discourse, as evidenced in “The History of Gonorrhea,” where “*linbing*” and “*baizhuo*” were explained as synonymous terms. This article provided detailed descriptions of various forms of gonorrhea, namely acute gonorrhea in men and women, and chronic gonorrhea in men and women (31); however, the interchangeable use of “*linji*” and “*linbing*” highlighted the instability of medical terminology during that period, reflecting an ongoing struggle to establish clear distinctions between related concepts.

The 1914 article “Warning to Shanghai Youth” exemplified the expanding semantic field of gonorrhea knowledge, juxtaposing gonorrhea and syphilis (collectively referred to as *hua liu bing*, a folkloric terminology of STIs), and underscoring the perceived severity of STIs (32). The 1916 article “On the Toxin of Gonococcus” further reflected a growing awareness of the broader health consequences associated with STIs and the need for public education. During this transitional phase, there was also a noticeable trend toward comparing Eastern and Western perspectives in discussions on gonorrhea. For instance, the 1914 article “On Gonorrhea in Chinese and Western Medicine” argued that “*linbing*” should not be equated with the TCM concept of “*linzheng*,” and thus the treatments of the two should differ (33).

### 3.3 The transformation stage (1920–1929)

The decade from 1920 to 1929 marked the transformation stage: the spread of gonorrhea-related knowledge became increasingly systematic, reflecting a shift from fragmented to structured information. A notable example was the 1927 article “A Brief Introduction to Gonorrhea,” which offered a detailed explanation of gonorrhea, covering its causes, pathogen, diagnostic methods, treatments, management protocols, and therapeutic approaches (34). During this period, the challenges of treating gonorrhea and its high transmissibility were emphasized, as was highlighted in “Gonorrhea, Formerly Known as *Baizhuo*”: “*Linzheng* is highly prevalent and extremely difficult to treat; it harms the health of the individual, and brings calamity to the family by causing childlessness (35).”

Another defining feature of this stage was the integration of sex education with public health initiatives, particularly those aimed at regulating behavior to reduce STI transmission. A typical example was the 1926 article “Sex Education and Prohibition,” which was published in *Shi Shi Xin Bao*. This article cited a study conducted by Dr. Meiromsky, which explored the link between alcohol consumption, sexual desire, and STI (including gonorrhea) prevalence. The author argued that prohibiting alcohol among the youth should be a core element of sex education in families and schools (36). This connection between sex education and public health policy reflected the evolving understanding of gonorrhea as not merely a medical issue but also a social one.

### 3.4 The stabilization stage (after 1930)

During the decade after 1930, the stabilization stage, the dissemination of gonorrhea-related knowledge became increasingly mature and refined. There was a noticeable shift in focus from individual health to public health. For example, the 1931 article “The Relationship Between Gonorrhea and Public Health,” which was published in *Wei Sheng Za Zhi*, introduced the concept of “gonorrhea carriers,” and called for the adoption of pre-marriage gonorrhea screenings, inspired by Western practices. Moreover, the article identified two primary reasons for the prevalence of gonorrhea—the public’s ignorance of its harmful effects and the lack of valid treatments among medical practitioners (37).

This phase also saw a transition from purely-medical knowledge to sex-oriented education. “Sexuality” became prominent within the gonorrhea-related discourse. For instance, a 1934 article was entitled “Knowledge on Sex: A Study of Gonorrhea.”

The discourse about gonorrhea also became more literary, reflecting an effort to make its knowledge more accessible to the general public. The rise of “medical vignettes” was a notable example—the 1938 article “The Terrifying Gonorrhea” conveyed two messages in the form of a short story: clean sexual practices are free from STIs, and visiting prostitutes inevitably leads to STIs (38). Such accessibility of gonorrhea-related knowledge was enhanced by an increasing number of articles published in magazines for the general public, such as *Sheng Huo Yue Kan* and *Chang Shou Zhou Kan*.

## 4 Discussion

After the founding of the People’s Republic of China in 1949, the understanding of gonorrhea underwent significant evolution, driven by medical advancements and increasing recognition of STIs as major public health concerns. Since 1989, gonorrhea has been listed as a legally notifiable infectious disease in the *Infectious Disease Prevention and Control Law* (39). By the late 20th century, it had been systematically incorporated into China’s national STD surveillance system, transitioning from a passive surveillance model to a sentinel surveillance system (40). Currently, China’s public health reporting system has evolved into a comprehensive Web-based surveillance framework (41). Eight STIs, including gonorrhea, syphilis, HIV/AIDS, genital warts, nongonococcal urethritis/cervicitis, genital herpes, lymphogranuloma venereum, and chancroid, are monitored (42). Additionally, China has actively engaged in international collaborations to enhance STI prevention and control efforts (43).

Interestingly, the reported incidence of gonorrhea in China is 26-fold lower than that in the U. S., with 6.83 cases per 100,000 people in China compared to 180 cases per 100,000 people in the US (44, 45), a disparity likely reflecting differences in diagnostic criteria, reporting mechanisms, cultural attitudes toward STIs, and prevention strategies. However, it is important to note that the successful control indicated by these statistics would not have be realized without the dissemination of gonorrhea-related knowledge from Western medicine to China outlined in this article.

This dissemination reflects a profound transformation in public perceptions of STIs across three key dimensions. First, there was a conceptual shift from understanding gonorrhea as a traditional illness caused by internal imbalances, a perspective rooted in TCM, to recognizing gonorrhea as a sexually transmitted disease, which is explicitly linked to external pathogens and sexual activities. Second, the framing of gonorrhea evolved from general medical education to sex-oriented education. Early efforts focused on the spread of clinical knowledge about symptoms and treatments, while over time, discussions increasingly integrated broader themes of sexuality, morality, and behavior regulation. Third, gonorrhea was acknowledged not merely as an individual ailment but as a societal issue. As it came to be collectively discussed and understood alongside other STIs, its knowledge construction demonstrates broader implications for public health, family stability, and societal well-being.

This case of gonorrhea serves as a compelling response to criticisms of TCM for its reliance on old literature and its perceived rigidity (46). It is true that Western medicine often seeks self-improvement through continuous innovation, while TCM frequently draws guidance from classical texts. However, this retrospective approach does not imply stagnation; instead, it reflects a distinct way of understanding health and disease, one that values historical wisdom and holistic perspectives while remaining open to novel theories. The interplay between TCM and Western medicine gave rise to a new terminology, encompassing new terms like *linjun* and *linbing*, which further facilitated Chinese people’s understanding of STI-related knowledge.

The historical evolution of gonorrhea in China provides valuable insights for contemporary STI prevention and control. First, it highlights the importance of using strategies to disseminate culturally-sensitive knowledge. The successful integration of Western medical concepts into Chinese public discourse demonstrates that effective communication requires linguistic and cultural contexts that ensure public understanding and reception. Second, it highlights the possibility of combining traditional wisdom and modern technology in the combat against STIs. While modern medicine emphasizes the elimination of pathogens, the holistic view of TCM, which stresses internal balance, might inspire complementary strategies for managing infections. Finally, public education remains critical. Raising public awareness and fostering precise perceptions of STIs are still essential for reducing transmission and restricting infection in diverse populations.

The historical trajectory of gonorrhea-related knowledge in China clearly reveals that mankind’s fight against STIs requires not only scientific advances but also cultural adaptability and public engagement. Whereas TCM emphasizes strengthening immunity and preventing disease through maintaining internal balance, the urgency of treating infections that may cause permanent damage without timely intervention highlights the indispensable role of Western medicine. In recent years, public health authorities in China have emphasized the importance of addressing antibiotic resistance in gonorrhea treatment (47). For STIs, enhancing immunity may be beneficial, but the prompt use of antibiotics remains the cornerstone of effective treatment. In this context, developing protocols that prioritize rapid diagnosis and treatment is essential to prevent irreversible harm, particularly in the face of rising antibiotic resistance among STIs.
